# Perspectives on innovative non-fertilizer applications of sewage sludge for mitigating environmental and health hazards

**DOI:** 10.1038/s44172-024-00298-x

**Published:** 2024-11-27

**Authors:** Elham H. Fini, Mohammadjavad Kazemi, Lily Poulikakos, Georgy Lazorenko, Vajiheh Akbarzade, Anthony Lamanna, Peter Lammers

**Affiliations:** 1 Arizona State University, 660 S. College Avenue, Tempe, AZ 85287 USA; 2 EMPA Materials Science and Technology, Ueberlandstrasse, 1298600 Dübendorf, Switzerland; 3 Novosibirsk State University, Pirogov Street, 2, Novosibirsk, 630090 Russia; 4 University of Doha for Science and Technology, 24449 Arab League St, Doha, Qatar

**Keywords:** Civil engineering, Environmental impact

## Abstract

As waste production increases and resources become limited, sewage sludge presents a valuable resource with potential beyond traditional land use and incineration. This review emphasizes exploring innovative non-fertilizer applications of sewage sludges and advocates for viewing wastewater treatment plants as sources of valuable feedstock and carbon sequestration. Innovative uses include integrating sewage sludge into construction materials such as asphalt pavements, geopolymer, cementitious composites, and masonry blocks. These methods not only immobilize heavy metals and mitigate environmental hazards but also support carbon sequestration, contrasting with incineration and land application methods that release carbon into the atmosphere. The review also addresses emerging technologies like bio-adhesives, bio-binders for asphalt, hydrogels, bioplastics, and corrosion inhibitors. It highlights the recovery of valuable materials from sewage sludge, including phosphorus, oils, metals, cellulose, and polyhydroxyalkanoates as well as enzyme production. By focusing on these non-fertilizer applications, this review presents a compelling case for re-envisioning wastewater treatment plants as sources of valuable feedstock and carbon sequestration, supporting global efforts to manage waste effectively and enhance sustainability.

## Abstract (web summary)

Ellie Fini and co-authors investigate innovative and often overlooked methods for managing sewage sludge. Their work emphasizes how these alternative approaches can effectively reduce environmental hazards, such as heavy metal contamination and carbon emissions, while also fostering the development of value-added products.

## Introduction

Sewage sludge is a waste resource with potential for broad application. The challenge is to create value from sewage sludge while protecting the environment and optimizing resource conservation. Conventional applications of sewage sludge provide some benefits but also cause concerns about contamination, necessitating innovative alternative methods that address environmental pollution while improving resource conservation and recycling^1^.

In 2017, worldwide production of sewage sludge from municipal water treatment plants reached approximately 45 million dry tons annually, with the United States, China, and Japan ranking as the top producers of sewage sludge globally^1,2^. The U.S. Environmental Protection Agency estimates that 4.5 million dry tons of treated sewage sludge were produced in 2021. Of this amount, 44% was applied to lands for soil enrichment, 42% was landfilled, and 13% was incinerated^3,4^. The land application of sewage sludges involves spreading them on the soil surface or incorporating or injecting them into the soil. Sewage sludges are applied to various sites, including agricultural lands, forests, mine reclamation sites, parks, and golf courses. Land application has been a common practice for decades and remains the most prevalent use for sewage sludges^5^. In the United States, 13 states allow the use of sewage sludges on food crops, while 17 states allow the application of sewage sludges only on residential and commercial lands. Maine is currently the only state with a ban on the land application of sewage sludges, and only a few states have legislation to manage per- and poly-fluoroalkyl substances in sewage sludges before applying it to lands^3^. In the European Union, 50% of sewage sludge is distributed on agricultural soils, 28% is burned, and 18% is landfilled^6,7^. Sludge fertilizer has been banned in Switzerland since 2006. Since then, incineration of sewage sludges has been planned; this was done because sludge contains many hazardous compounds and pathogenic organisms from industry and households^8^. In Russia, sewage sludge management practices have historically focused on landfilling, but recent initiatives are promoting the use of sludge in agriculture and energy recovery through incineration and biogas production. In Australia, more than half of sewage sludges are applied to lands^9^. In the Middle East, Saudi Arabia leads the sewage sludges market with a projected compound annual growth rate of 6% during 2022–2027^10^. Generally, 20% to 50% of the operational expenses of wastewater treatment plants are allocated to sludge management and disposal^11^. This underscores the pressing need for efficient and economically viable strategies for recycling or disposing of sewage sludges.

Sewage sludges represent a valuable resource that is rich in organic matter, nitrogen, phosphorus, micronutrients, proteins, cellulose, and various trace elements^12–15^. These properties render sewage sludges beneficial for diverse applications such as agriculture, silviculture, horticulture, and land reclamation^16,17^. Sewage sludges’ high caloric value, at 12 to 18 MJ per kg, positions them as a potential energy source^18^; however, raw sewage sludge contains elevated levels of viruses, pathogenic bacteria, parasite eggs, heavy metals, microplastics, and polyfluoroalkyl substances/perfluorooctane sulfonic acid (PFAS/PFOS), necessitating careful management^19–23^. Studies have revealed associations between exposure to specific perfluoroalkyl and polyfluoroalkyl substances and a variety of health effects, including but not limited to liver disease, kidney disease, and cancer^24^. Table 1 shows the concentration range of various chemicals in the dry weight of sewage sludge. While agricultural use and incineration are common methods of repurposing sewage sludge, these methods can create additional environmental and health problems. Burning sewage sludge releases noxious gases that harm human health and ecological systems^25^. Precipitation events can carry combustion byproducts from the air into water bodies, polluting aquatic ecosystems and intensifying environmental issues^2^. Consequently, the safe, cost-effective, and environmentally friendly treatment of sewage sludge has become an urgent global challenge requiring innovative solutions.

**Table 1:** Concentration range of contaminants found in the dry weight of sewage sludge

Chemical name	Range of concentration (min–max)	Units	References
2-(N-Ethylperfluorooctanesulfonamido) acetic acid	Not detected–11	µg/kg	^159^
2-(N-Methylperfluorooctanesulfonamido) acetic acid	18–23	µg/kg	^159^
alpha-Solanine	0.547–183.01	ng/mL	^160^
Berberine	10.044–27.874	ng/mL	^160^
Bromide	1.215–1.22	mg/L	^161^
Doxepin	0.082–0.55	ng/mL	^160^
Fentanyl	0.041–0.33	ng/mL	^160^
Hydromorphone	0.254–0.354	ng/mL	^160^
Hydroxychloroquine	6.858–67.631	ng/mL	^160^
Levorphanol	0.877–2.01	ng/mL	^160^
Losartan	1.655–7.758	ng/mL	^160^
Methadone	0.442–0.88	ng/mL	^160^
Perfluorohexadecanoic acid	~ 0–1.6	µg/kg	^162^
Bisphenol A	100–1300	ng/g	^163^
Perfluorodecanesulfonic acid	~ 0–3.2	µg/kg	^162^
Potassium	486–9776	ppm	^162,164^
Azithromycin	0.06–21.276	mg/kg	^160,165^
Carbamazepine	~100	µg/kg	^166^
Ciprofloxacin	1.83–1	mg/kg	^165,166^
Cocaine	0.459–1.423	ng/mL	^160^
Codeine	0.069–0.232	ng/mL	^160^
Gemfibrozil	~2	µg/kg	^166^
Ibuprofen	~140	µg/kg	^166^
Miconazole	10,382	ng/g	^167^
Naproxen	~25	µg/kg	^166^
Ofloxacin	~148	ng/g	^167^
Oxycodone	0.067–0.134	ng/mL	^160^
Perfluorobutanesulfonic acid	0.4–41.9	µg/kg	^159,162^
Perfluorobutanoic acid	0.6–6.5	µg/kg	^162^
Perfluorodecanoic acid	1.4–20.5	µg/kg	^159,162^
Perfluorododecanoic acid	1–7.7	µg/kg	^159,162^
Perfluoroheptanoic acid	0.15–6.5	µg/kg	^159,162^
Perfluorohexanesulfonic acid	0.42–15	µg/kg	^159,162^
Perfluorohexanoic acid	0.5–61	µg/kg	^159,162^
Perfluorononanoic acid	0.7–8.1	µg/kg	^159,162^
Perfluorooctanesulfonic acid	2.6–88.5	µg/kg	^159,162^
Perfluorooctanoic acid	1.2–26	µg/kg	^159,162^
Perfluorotetradecanoic acid	0.6–3.3	µg/kg	^159,162^
Perfluorotridecanoic acid	0.1–2.5	µg/kg	^159,162^
Perfluoroundecanoic acid	1–8	µg/kg	^159,162^
Triclocarban	~86	ng/g	^167^
Triclosan	111–8240	ng/g	^168,169^
Iron	2000–38,000	mg/kg	^170–172^
Magnesium	430–1260	ppm	^162^
Manganese	100–2621	mg/kg	^170,173,174^
Silver	2.5–60	mg/kg	^175^
Copper	75.8–801	mg/kg	^176–178^
Zinc	300–7500	mg/kg	^173,176^
Chromium	225–900	mg/kg	^171,178,179^
Nickel	8.6–420	mg/kg	^174,180^
Lead	30–430	mg/kg	^172,174^
Cadmium	0.83–3.0	mg/kg	^177,181^
Mercury	0.1–1.1	mg/kg	^182^
Arsenic	9.9–56.1	mg/kg	^181,183^

Investigations into biofuel extraction from sewage sludges have primarily focused on using processes such as pyrolysis^26,27^, hydrothermal liquefaction^28,29^, and transesterification^30^. These processes are naturally energy-intensive and increasing the temperature during the process can result in the production of nitrogen- or sulfur-containing pollutants, such as HCN, NH_3_, and H_2_S. These pollutants can be introduced into the final products (char, gas, and oil) and cause a degradation in product quality^31^. Concurrent studies examining sewage sludges’ use as a fertilizer delve into various purification techniques and address concerns related to microplastics; however, these studies often overlook the broader risks associated with additional contaminants, such as PFAS/PFOS. Table 2 summarizes the advantages and disadvantages of using sewage sludges as fertilizer. It is noteworthy that the prevalence and ensuing complexities of soil application stem from the conventional practice of providing sewage sludges at minimal or no expense. Despite the availability of sewage sludges, farmers frequently opt for commercial fertilizers due to potential risks to crop quality.

**Table 2:** Advantages and disadvantages of the application of sewage sludge as fertilizer

Advantages	Disadvantages
• Sewage sludge can boost plant growth by providing nutrients such as nitrogen and phosphorus, and having organic matter can improve soil structure, water retention, and aeration^184^. • It reduces the need for synthetic fertilizers by recycling nutrients from wastewater^185^. • Cost-effectiveness^186^. • Sewage sludge can improve soil structure and increase water retention in sandy soils or drought-prone areas^187^.	• Compared with mineral fertilizers, sewage sludge has a lower nutrient content^188^. • High concentrations of pathogens and heavy metals in sewage sludge, including zinc, copper, and chromium, exceed thresholds, posing ecological risks. This can lead to metal contamination in crops, raising health concerns. It can also harm soil microorganisms, lowering agricultural soil quality^188–190^. • Sewage sludge can decrease the capacity for seed germination and decrease the length of roots of some plants^191^. • The effectiveness of sewage sludge depends on the origin (urban or industrial), application rate, and plant type^192^. Some sludges are unsuitable for direct application due to high Zn or Ni content, and many sludges are improper for fertilizer production due to Cd, Cr, and/or Ni content^193^. • Ingestion of sewage sludge containing arsenic and cadmium poses carcinogenic hazards, particularly for children^189^. • The safe application period for sewage sludges is approximately 17 years, during which toxic metal accumulation does not exceed the limits and the risk to human health is minimal^194^.

This review seeks to illuminate the untapped potential of sewage sludge, commonly known as sewage sludges, extending beyond the conventional applications in agriculture and fuel generation. The conventional uses not only suffer from a lack of cost-effectiveness but also pose inherent risks to both human health and the environment. Exploring alternative applications for sewage sludge presents an opportunity to uncover more sustainable and innovative solutions addressing these concerns. This broader perspective on sewage sludges opens up avenues for research and development in areas traditionally overlooked, fostering a more comprehensive understanding of the potential benefits and challenges.

### Non-conventional applications of sewage sludge

#### Direct applications of sewage sludge and sewage sludge ash

One possible application of sewage sludges is in the development of cost-effective and environmentally sustainable materials for the construction sector. The primary constituents found in sewage sludge are Al_2_O_3_, SiO_2_, and CaO, rendering it chemically analogous to clay^32^. This gives sewage sludge a theoretical base in the field of ceramic and brick preparation^33^. The use of sewage sludge ash as a partial substitute for natural clay has been shown to be a viable strategy for the production of more environmentally sustainable bricks. This feasibility arises from its chemical similarity to clay, enabling attainment of the desired densification, increased strength, and reduced absorption through heat treatment^34^. The resulting material exhibited increased porosity, which can be attributed to a higher concentration of hematite in sewage sludge. A more carefully controlled production procedure is necessary to prevent detrimental effects on mechanical qualities^35^. Bricks having a replacement ratio of clay to sewage sludge ash of less than 20% demonstrate increased compressive strength compared to conventional bricks; this is reflected in higher density and reduced shrinkage; however, when exposed to fire, these bio-modified bricks also exhibit greater mass loss. This increased mass loss is due to the presence of substantial amounts of organic matter, which decomposes at high temperature. At high temperatures, components such as calcium carbonate reduce to calcium oxide and in the process release CO_2_^36^. The findings of environmental risk assessments indicated that the incorporation of sewage sludge containing metal oxide in the brick’s composition resulted in the solidification of heavy metals. Solidification involves a meticulous combination of these materials with binders and reagents in construction materials aimed at minimizing the leaching of contaminants. This is achieved by reducing the mobility of metals through their physical encapsulation in a low-permeability material^37^. This solidification process effectively immobilized the heavy metals, thereby preventing their release into the surrounding environment^33^. Specifically, it was observed that the solidification process completely eliminated cadmium (Cd) leachate, reduced the leachate of arsenic (As), lead (Pb), and zinc (Zn) to very low levels, and reduced the leachate of copper (Cu) and nickel (Ni) to moderate levels^38^. The observed variations in the efficacy of heavy-metal immobilization through solidification can be directly attributed to specific factors. These include the initial concentration levels of metals in the sludge, the chemical state of the metals, and their interactions with the clay components of bricks, particularly in relation to the pH environment. The leaching of copper and nickel was moderately reduced, likely because of their different ionic forms and the extent of their chemical binding within the brick matrix^38,39^. Also, after the washing of phosphorus from sludge for the purpose of using the phosphorus as fertilizer or food additives, it was noticed that an increase in the use of phosphorus-depleted sludge had a negative impact on the compressive strength of bricks. There was a 39% reduction in compressive strength when 50% of the clay was replaced with sewage sludge, compared to the control bricks. However, this modification led to an improvement in the thermal conductivity of the bricks, with 50% clay substitution showing 21% better thermal conductivity^40^. Departing from this replacement ratio leads to a substantial reduction in density, since the generation of open porosity increases by approximately 13%. However, it should be noted that regardless of the replacement ratio, this modification also leads to an increase in the water-adsorption potential of the bricks^40^. The impact of sewage sludge on the extrusion process of ceramic bricks was shown to be detrimental, as an increase in sewage sludge content necessitated a higher water content for optimal performance. The modification of clay blends with sewage sludge changed their viscoelastic properties by altering the ratio between the loss modulus and the storage modulus. This modification led to a higher proportion of elasticity in ceramics that were modified with sewage sludge^41^.

Portland cement is a construction material known for its high pollution levels. The presence of pozzolanic reactivity in sewage sludge ash makes it a viable candidate for partially substituting Portland cement in construction applications^32^; however, the use of sewage sludge ash in regular concrete hindered the hydration process and resulted in lower compressive strength when compared to traditional concretes^42,43^. It is recommended that regular concrete mixed with sewage sludge (particularly when the sludge concentration exceeds 2% by weight of cement) should primarily be used for low- or medium-strength applications such as pavements, walkways, non-structural construction, pre-cast concrete products, and mass concrete bodies. This is because the concrete has limited hydrated compounds and bond development in its matrix^42^. Life-cycle cost analysis has shown that a 20% replacement of Portland cement by sewage sludge ash achieves a strength-normalized unit cost of concrete of 1.9 $/m^3^/MPa. This marks a high reduction of 52.5% compared to conventional concrete^32^. Research has indicated that when sewage sludge ash was used in a cementitious matrix, there were substantial reductions in the leaching of heavy metals: chromium by 81.82%, copper by 38.59%, zinc by 90.74%, lead by 87.45%, and nickel by 56.17%. These reductions were attributed to the dilution and stabilization effects provided by the binder materials^44^. Additional studies demonstrated the potential of using calcined sewage sludge as a partial replacement for cement in concrete. Incorporating this material by 30% of the weight of cement led to a 2.72% reduction in costs and a 26.39% decrease in carbon emissions. Moreover, this approach effectively stabilized heavy metals, particularly manganese, by transforming soluble manganese into a more stable, less soluble oxide form through high-temperature calcination, which minimized its leachability^45^. Prabhakar et al. used sewage sludge ash as a substitute for both cement and aggregate in the production of mortar, with potential applications in seawall construction. They showed that mortars with sewage sludge ash can be environmentally safe, with leachates not affecting microalgae growth; however, some toxicity effects were observed in embryos of sea urchins, indicating potential risks to sensitive marine species. Leachate tests confirmed that while the mortar with sewage sludge ash is generally non-toxic to microalgae, the presence of certain heavy metals such as chromium, nickel, and arsenic in the leachates could pose ecological risks to marine environments^46,47^.

**Fig. 1:**
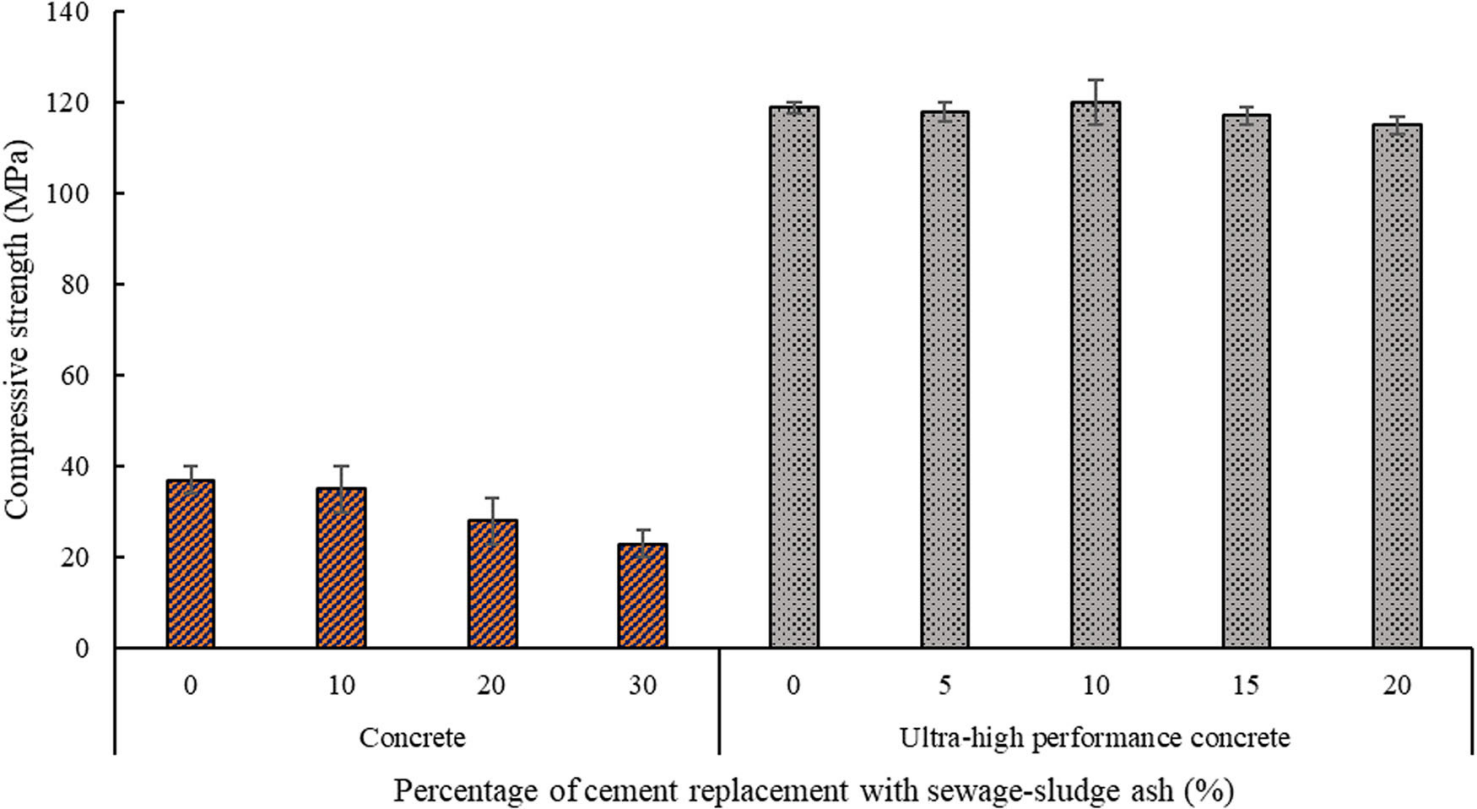
The compressive strength of concrete and ultra-high-performance concrete at different percentages of cement replacement with sewage sludge ash (data secured from refs. ^32,48^).

The porosity and elevated water-adsorption properties of sewage sludge ash make it advantageous as a curing agent for use in ultra-high-performance concretes^32^. With increasing concentrations of sewage sludge ash up to 10%, ultra-high-performance concretes’ flowability and hydration-heat release diminish, but long-term strength development increases marginally^48^. Figure 1 shows the compressive strength of normal concrete and ultra-high-performance concrete containing different dosages of sewage sludge ash^32,48^. Including sewage sludge ash in ultra-high-performance concretes leads to alterations in the microstructure and phase composition of the mixture by increasing the Si/Ca, Al/Ca, and Fe/Ca ratios of the calcium-silicate-hydrate in the concrete mixture. A cradle-to-gate life-cycle assessment demonstrated that substituting 8 wt.% of cement with sewage sludge ash in ultra-high-performance concrete decreases the consumption of non-renewable fossil fuels (fossil-fuel depletion indicator) by 6.2% and reduces greenhouse gas emissions (indicator of global-warming potential) by 8.2%. These indicators—fossil-fuel depletion and global-warming potential—serve as quantifiable measures of the environmental impact, assessing the sustainability benefits of incorporating sewage sludge ash^32^. Sewage sludge ash can also be used to produce zeolite crystals with a crystalline structure similar to commercial zeolites, with a relatively larger percentage of crystal water and a more gradual release of crystal water during heating. Producing an asphalt mixture using these bio-based zeolites has shown a reduction of 35.6% to 50% in the cost per kilometer of warm-mix asphalt^49^.

Sewage sludge ash can be used as a precursor of low-carbon magnesia-based cements or as alkali-activated materials in geopolymers. The dissolved phosphorus in sewage sludge ash improves water resistance and compressive strength in magnesium oxychloride cement, and the amorphous phase containing Mg, Si, and Cl gives it high water stability^50,51^. Geopolymerization of sewage sludge ash with ground granulated blast-furnace slag^52^ and metakaolin^53^ provides effective immobilization of radionuclides and heavy metals in cured products because of a dense and compact 3D-network structure. Due to its limited reactivity in alkaline environments, sewage sludge ash combined with precursors rich in silica, alumina, and calcium usually provides higher mechanical strength and greater durability than sewage sludge alone^54,55^.

Sewage sludge^56^ or its derivatives hydrogels^57^, biochar^58,59^, and ash^60^ serve as valuable precursors for producing sorbents aimed at removing contaminants from air, soil, and water^61^. Sewage sludge-based sorbents (biochar, activated carbon, and ash) are produced through thermochemical processes that enhance their porous structure and surface area, making them effective for adsorbing pollutants^62^. Also, these materials contain metal oxides that facilitate the removal of contaminants through adsorption and chemisorption, proving particularly valuable in capturing heavy metals, organic pollutants, and greenhouse gases^63–66^. Additionally, sewage sludge-derived hydrogels excel in water purification by swelling to retain large volumes of water while effectively adsorbing contaminants^57^. Table 3 summarizes successful studies on the use of sewage sludge-based sorbents for decontaminating various pollutants from air, water, or soil.

**Table 3:** Decontamination efficiency of sorbents based on sewage sludge

Sorbent material	Target contaminant	Target environment	Performance and properties	Ref
Alum sludge	hydrogen sulfide (H_2_S)	Landfill biogas	Alum sludge showed a high adsorption capacity for H_2_S, with the capacity reaching up to 374.2 mg of H_2_S per gram of sorbent at the lowest flow rate (3 L/h) and highest bed depth	^56^
Cerium-doped aluminum sludge hydrogel	Phosphate	Wastewater	• Cerium-doped aluminum sludge hydrogel achieved maximum phosphate adsorption of 20.36 mg P/g, marking a 292% increase over raw sludge. • The phosphate adsorption process was determined to be chemisorption. • The adsorption process was pH-sensitive, with a pH of 4 as the most effective.	^57^
Hydrogel loaded with sewage sludge ash	Chromium, phosphorous, and ammonium	Wastewater	• High pollutant removal efficiency: chromium removal 96.9–97.4%, phosphorus removal 97.7–99.6%, and ammonium removal 76.8–77.9%. • Sewage sludge ash-loaded hydrogel achieved an average water flux value of 1.95 to 2.02 liters per square meter per hour, nearly double that of normal hydrogel. • The hydrogel doped by sewage sludge ash demonstrated greater swelling properties, with an optimal critical content sewage sludge ash of 2.5 wt%, resulting in a 12.9% improvement over the base hydrogel. • Adding attapulgite clay and poly(N-isopropylacrylamide-co-acrylic acid) to hydrogel loaded with sewage sludge ash further increased surface area and additional active sites on sludge, resulting in better sorption function.	^141,142,195,196^
Biochar derived from sewage sludge	CO_2_, PFAS, arsenic, bisphenol A, tetrabromobisphenol A	Wastewater, soil, and air	• Biochar derived from sewage sludge was reported to have a CO_2_ adsorption capacity of 0.212 mmol/g to 0.415 mmol/g, depending on production temperature. • Biochar derived from sewage sludge has been shown to reduce leachate of PFAS from the soil by 95%, while wood-based biochar and commercial active carbons showed less than 42.4%. • Arsenic adsorption onto activated carbon is faster than onto pyrolyzed sludge, with a maximum capacity of 71 μg/g for the sludge and 229 μg/g for activated carbon. • Biochar derived from sewage sludge has been reported to have a maximum tetrabromobisphenol A adsorption capacity of 87.02 mg/g at 30 °C and pH 7.5. • Modifying biochar with potassium hydroxide enhances its hydrophobic properties, improving the adsorption of hydrophobic contaminants such as BPA by up to 30% to 170 mg/g.	^58,59,197–201^
Sewage sludge ash	Hydrogen sulfide (H_2_S)	Landfill biogas	• Sewage sludge ash helps the pH stabilization in a basic range for better H_2_S dissociation into HS− and chemisorption. • Adding 20% active carbon to sewage sludge ash increases surface area for effective chemisorption kinetics.	^60^
Sludge-based activated carbon	Mercury, PFAS, pharmaceutical, and personal care products such as ibuprofen, 2-hydroxy-ibuprofen, naproxen, triclosan, triclocarban, and bisphenol A	Flue gas, wastewater	• Fe-containing sewage sludge activated with sulfuric acid showed a mercury adsorption capacity of 14.8 mg/g from the gas flow. • Sludge-based activated carbon is produced at a 60% lower price compared to commercial activated carbon, which costs over $3 per kg. • For PFAS removal, sludge-based activated carbon demonstrated similar or better efficiency compared to commercial AC, with over 91% PFAS removal rate. • Sludge-based activated carbon was successful in removing 91.6% to 99.8% of ibuprofen, 2-hydroxy-ibuprofen, bisphenol A, and naproxen at a concentration of 1 g/L and 100% at a higher concentration of 10 g/L. Antimicrobial compounds such as triclosan and triclocarban were nearly 100% removed at both dosages of sludge-based activated carbon.	^64,202,203^

#### Applications of materials recovered from sewage sludge

The recovery of materials from sewage sludge has become a valuable method for recycling them. Recovered materials include oils^67^, metals such as gold, copper, nickel, zinc, chromium, and cadmium^68–70^, and bio-based polymers such as cellulose^71^ and polyhydroxyalkanoates^72,73^. Several isolated or mixed biowaste has shown effective oil recovery using hydrothermal liquefaction at 300–350 °C^74^, producing oil with thermal stability, viscoelastic thermoplastic behavior, and high compatibility with petroleum-based asphalt binder^75^. This recovered oil can be used to partially replace petroleum-based asphalt binder and even improve the properties of aged petroleum-based asphalt binder^76^. The bio-based asphaltenes found in sewage sludge oil have a higher content of heteroatoms than normal asphalt, which affects properties such as acidity, polymerization, viscosity, and mechanical behavior upon aging; they also were shown to have lower polarizability than petroleum-based asphalt^77^. Additionally, the presence of nitrogen in bio-based asphaltenes alters π-π interactions^78^, potentially improving asphalt’s rheological properties and cracking resistance^79,80^. It has been shown that a 10% replacement of asphalt binder with sewage sludge oil not only softens the asphalt binder without compromising its cohesive strength but also reduces the emission of harmful gases such as nitrogen oxides and sulfides without posing a risk of leaching heavy metals from pavement^81^.

Up to 40% of the suspended solids in influent wastewater are attributed to cellulose fibers from toilet paper^82^. Alkaline treatment of sewage sludge, which is rich in cellulose primarily from disintegrated toilet paper, has been shown to recover more than 85% of the cellulose content^83^. However, studies have shown that cellulose recovery varies depending on the type of sludge, with primary sludge yielding the highest content at 7.1% of total solids (largely due to cellulose from toilet paper); excess secondary sludge and dewatered secondary sludge show lower levels at 2.3% and 2.6%, respectively, and digested types exhibit the lowest recovery at 1.0% to 1.1%^82^. This recovered cellulose can be reused in diverse applications; for instance, recovered cellulose fibers have been used as the fortification of lime-based mortars for nonstructural applications. The recovered cellulose fibers lightened the mortars but improved their flexural strength and residual compressive strength^84^. Polyhydroxyalkanoates (PHAs) are biodegradable polyesters that can be used as raw material for the production of bio-based biodegradable plastics (bioplastics). Sewage sludge is a cost-effective source for extracting crude polyhydroxyalkanoates, with an average cost of US$1.76/kg PHA-crude, regardless of treatment technology or plant size^85^. Other dissolved organic substances such as alginate-like biopolymers and amyloid-fibrils-like adsorbents that have been recovered from sewage sludge have been found to be effective adsorbents of heavy metals such as Cd^2+^, Ag^+^, Ni^2+^, and Cu^2+^ ^86,87^. Specifically, alginate-like biopolymers demonstrated effectiveness in Cd^2+^ adsorption, while amyloid-fibrils-like adsorbents were found to be effective for Ag^+^, Ni^2+^, and Cu^2+^. Optimal conditions for Cd^2+^ adsorption with alginate-like biopolymers included a dosage of 7.9 g d.m./L, a pH range of 4–8, and an equilibrium time of 60 min, with carboxyl and hydroxyl groups identified as key functional groups involved in Cd^2+^ adsorption^86^. Additionally, amyloid-fibrils-like adsorbents with higher molecular weight showed better performance for Ag^+^ because of the higher affinity of Ag^+^ to macromolecules, while smaller molecules provided more sorbing sites for Ni^2+^ and Cu^2+^^87^. The Electro-Fenton (EF) process offers a promising method for wastewater treatment, leveraging electricity to produce hydrogen peroxide and ferrous ions. These components react to generate hydroxyl radicals, potent molecules adept at decomposing various organic contaminants in water^88^. Enhancing this process, catalytic cathodes—recently developed from coagulant sludge—show excellent electrical conductivity and catalytic action for the oxygen-reduction reaction, thus improving the efficiency of pollutant degradation^89^. Beyond wastewater treatment, catalysts are instrumental in the recycling of plastics, facilitating the breakdown and transformation of plastic waste into valuable products. In pyrolysis and similar processes, catalysts reduce the required reaction temperature and accelerate the chemical decomposition of plastics, making the process more energy-efficient and effective. Specifically, sewage sludge char has emerged as an effective catalyst for the selective production of aromatics during the pyrolysis of mixed waste plastics. The efficacy of this catalytic material is largely dependent on its ash content, which varies based on the source of the sludge. The carbonization of sewage sludge produces ash that acts as active sites for catalysis, with its increased surface area providing more exposure for active components, thereby enhancing catalytic activity^90^. An inexpensive, environmentally friendly corrosion inhibitor for steel has been developed using rapid hydrolysis of sludge proteins to develop a hydrolysate containing several amino acids such as d-aspartic acid, glutamic acid, glycine, and alanine; the adsorption of this hydrolysate onto the surface of steel could effectively protect the steel from corrosion in acid media. However, it is noteworthy that the inhibitor’s functionality experienced a decline as the temperature of the medium increased^91^. Latosińska et al. used sewage sludge ash to produce zeolite X using the fusion method and reported the performance of zeolite X in treating landfill leachate from the Promnik landfill site. The modified sewage sludge ash containing zeolite X effectively removed heavy metals such as copper and lead from landfill leachate, with copper removal reaching near-complete levels under certain conditions; it also reduced the chemical oxygen demand and organic compounds, showcasing its robust capability in purifying complex landfill leachates^92^.

Sewage sludge is a source of phosphorus (P); there is a growing emphasis on its recovery and reuse due to depleting natural reserves of phosphorus^93^. Currently, phosphorus recovery from sewage sludge can be achieved with a recovery rate of about 32%–98% with 5 to 25 grams of phosphorus per kilogram of dry sludge, varying greatly based on the technology and local conditions^94–101^. The average costs for phosphorus recovery are estimated to range from 2 to 8 euros per kilogram^102,103^, whereas the extraction costs for rock phosphate are cheaper, at just 0.035 to 0.05 euros per kilogram. However, for every kilogram of phosphorus that is not dumped into the environment by being recovered, the damage prevented or the environmental benefit generated equals €42.74^104^. A case study shows that incorporating environmental benefits turns a potential annual net loss of 76,762.05 euros into a net gain of 94,198.17 euros, underscoring the economic and environmental value of recovering phosphorus from sewage sludge^104^.

Recovered phosphorus can be used in the production of fire-safety products. Phosphorus compounds are integral in manufacturing the flame retardants used in textiles, plastics, and building materials to enhance fire resistance. Additionally, phosphorus is used in the metal finishing industry, where phosphoric acid, derived from recovered phosphorus, is used for the surface treatment and cleaning of metals, helping to prevent corrosion and improve the adhesion of paint on metallic surfaces. These applications underscore the versatility and industrial importance of recovered phosphorus, extending its benefits beyond traditional agricultural uses.

**Fig. 2:**
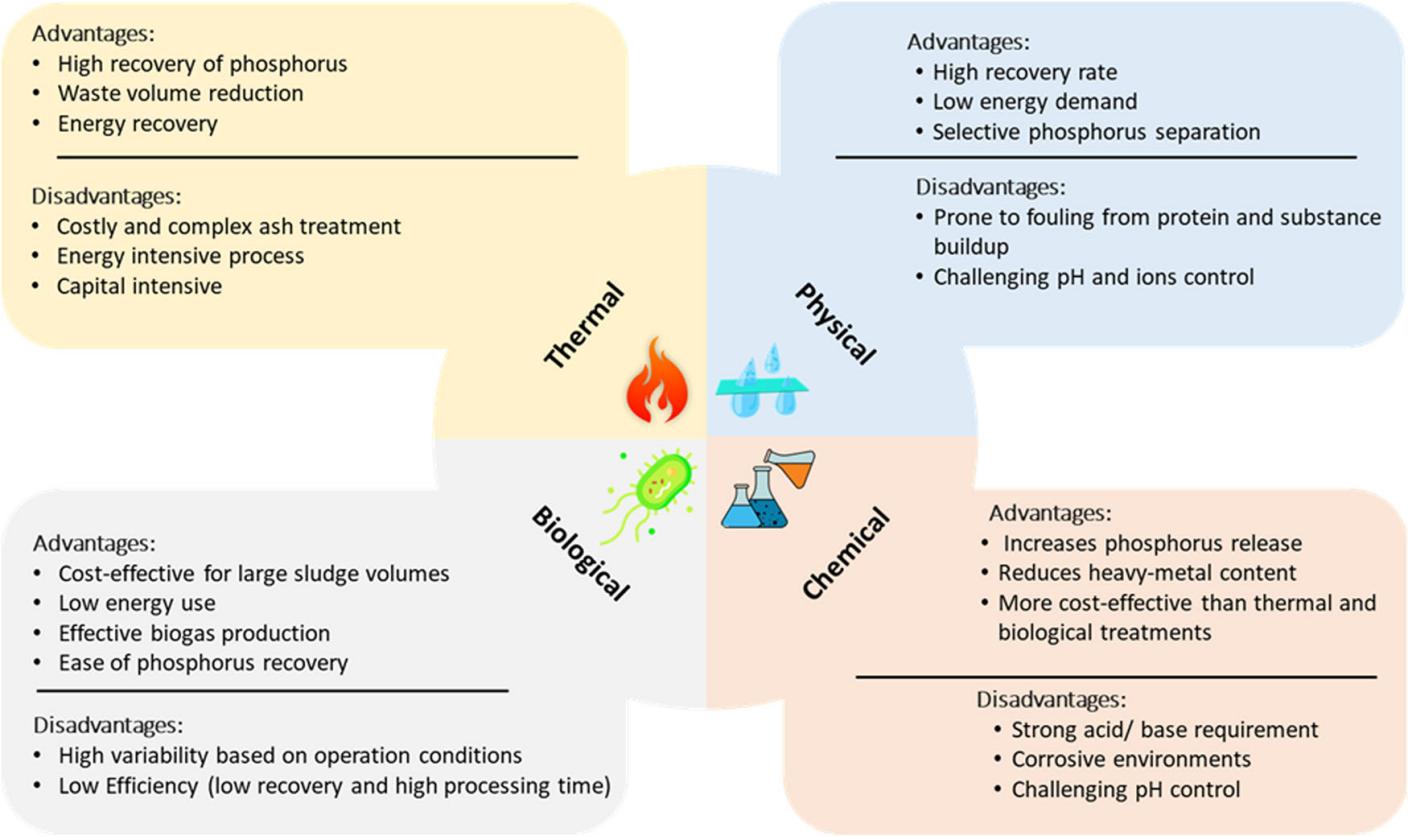
Comparison of four general technology categories for phosphorous recovery from sewage sludge.

Four general approaches are available to recover phosphorus from sewage sludge: thermal treatment^105,106^, physical treatment, chemical treatment, and biological treatment. Thermal treatment involves applying high temperatures to sewage sludge to convert it into ash or biochar, where phosphorus is concentrated^105^. Methods include hydrothermal carbonization^93,107,108^, pyrolysis^93,109^, and gasification^110,111^. The phosphorus can then be chemically extracted from the ash or biochar for reuse. Physical treatment focuses on mechanical processes to separate phosphorus-rich fractions from sewage sludge^112^. The processes include electrodialysis^113,114^ and membrane filtration^112,115^. These methods concentrate phosphorus into a smaller volume that can then be further processed for recovery^112^. Chemical treatment involves adding chemicals to sewage sludge to precipitate phosphorus compounds^116^. Common methods include the addition of coagulants such as iron or aluminum salts, which form insoluble phosphorus compounds that can be removed and processed^117,118^. Acid or alkaline leaching is also used to dissolve phosphorus^119^. Biological treatment leverages microbial activity to remove and recover phosphorus from sewage sludge^120^. Enhanced biological phosphorus removal processes encourage certain bacteria to accumulate phosphorus within their cells. The phosphorus-rich biomass can then be harvested^120,121^. Figure 2 summarizes the advantages and disadvantages of these methods^93,98,105–114,116–129^.

#### Production of enzymes from sewage sludge

**Fig. 3:**
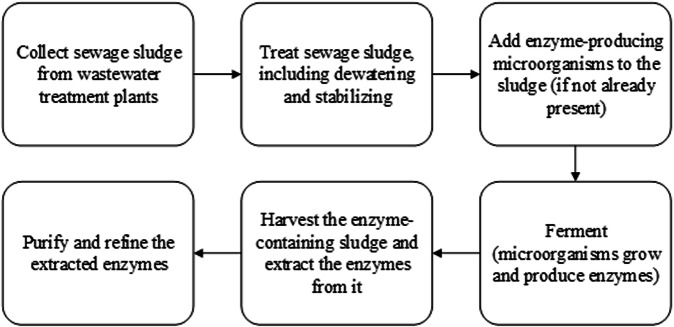
Overview of using sewage sludge for enzyme production.

Sewage sludge is a valuable source of microbial diversity that can be used to produce enzymes^130^. These enzymes have a wide range of applications, including use in detergents, food, pharmaceuticals. Traditional methods of producing enzymes using synthetic media can be very expensive because of the need for expensive and carefully formulated chemical components to provide the necessary nutrients for microbial growth and enzyme production^131^. By shifting toward more sustainable practices, the use of sewage sludge as a raw material can provide a cost-effective alternative. Sewage sludge contains valuable nutrients that can serve as an alternative media for enzyme production^132^. Figure 3 shows the general process of using sewage sludge for enzyme production.

Robledo-Mahón et al. aimed to discover microorganisms with biotechnologically relevant enzymatic activities during the composting of sewage sludge. They isolated 40 fungal and 128 bacterial strains, finding diverse microorganisms producing enzymes including amylases, proteases, cellulases, and more. Genera such as *Aspergillus*, *Bacillus*, and *Pseudomonas* showed potential applications. The study suggests composting of sewage sludge is a sustainable method for obtaining valuable enzymes from waste biomass^133^. Research on yeasts from diverse wastewater treatment systems, focusing on the production and distribution of extracellular enzymes, found that among 257 isolated yeasts, 73 demonstrated the capacity to produce lipase, protease, manganese-dependent peroxidase (MnP), and lignin peroxidase (LiP). These enzyme-producing yeasts, exhibiting a wide phylogenetic distribution, displayed competence in decolorizing synthetic dyes through effective biodegradation or biosorption mechanisms^134^. Drouin et al. explored the potential of *Bacillus licheniformis* proteases as valuable products from the fermentation of wastewater sludge. Ultrasonication and heat treatment enhanced the solubilization of the organic matter in the sludge, making wastewater sludge a more effective and nutrient-rich medium for formation of *Bacillus licheniformis*. Fermentation by *Bacillus licheniformis* yielded proteases with high activity (1.67 g/L, specific activity of 19.8 U/mg). These proteases, characterized by stability and versatility, showcased the potential for high-value applications^135^.

#### Risk of toxicity from products derived from sewage sludge

**Fig. 4:**
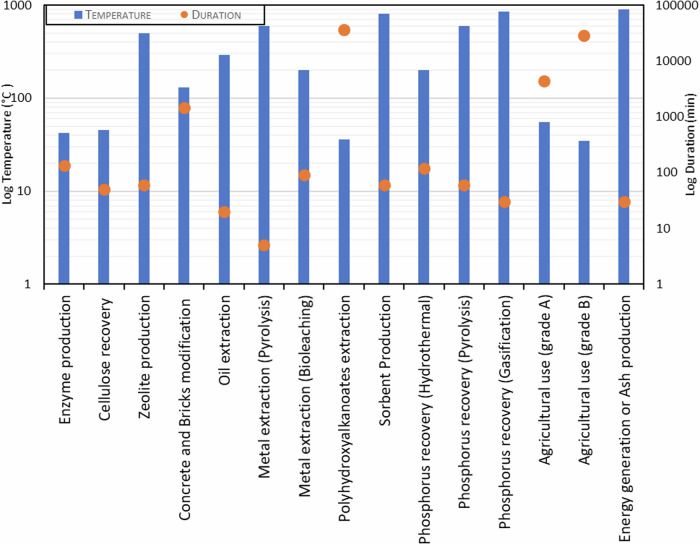
Temperature and duration of heat treatment involved in novel and conventional applications of sewage sludge (Data source: refs. ^3,4,42,49,69,71–73,82,83,92,113,133–135,141–143^).

As mentioned, sewage sludge contains pathogens and pyrogens. Typical hygienization of sewage sludge at 70 °C does not eliminate spores, pyrogens, or pathogens^136,137^. DNA is denatured at 90 °C. Therefore, high-temperature treatments or converting the sludge to char or ash will remove bacteria, fungi, virus spores, parasites, and antibiotic-resistance genes from the final product, resulting in far less pollution and the associated public health concerns compared to the direct land application of sewage sludge^138^. Other pollutants found in sewage sludge require higher temperatures to degrade. Microplastics start to degrade at around 100 °C, depending on the type of plastic^139^. For other contaminants, such as perfluorooctanoic acid (PFOA), degradation starts at temperatures around 200 °C. However, perfluorooctanesulfonic acid (PFOS) typically requires a much higher temperature ( ≥ 450 °C) to degrade^140^. Metal contaminants such as mercury require even higher temperatures, in excess of 500 °C^140^. Figure 4 was developed based on data retrieved from the literature pertaining to temperature and duration of heat treatment for various applications of sewage sludge^3,4,42,49,69,71–73,82,83,92,113,133–135,141–143^.

Conventional applications of sewage sludge, such as agricultural use and energy generation through incineration, present health and environmental risks because of inadequate heat treatment or emission of toxic gases to the atmosphere^144^. Preprocessing of sewage sludge before agricultural use does not involve heat treatment prior to its usage in agricultural fields; thus, most of the contaminants remain in the sludge, directly exposing the soil and plants to pathogens and pollutants. Although incineration involves high temperatures, it is associated with high emissions of harmful pollutants, contributing to degradation of air quality and posing health risks to nearby communities.

In contrast, novel applications of sewage sludge use higher temperatures that effectively reduce health risks by destroying pathogens and degrading contaminants. For instance, the use of sewage sludge in concrete and bricks not only involves temperatures above the threshold required to denature DNA (90 °C) but also integrates binders that stabilize the material and prevent leachate of contaminants. This application not only secures the sludge but also contributes to safer construction materials. Hydrothermal liquefaction of sewage sludge into oil has been reported to reduce harmful pathogens and toxins, minimize landfill use, and transform over 70% of the organic content into bio-oil. This process captures carbon in a liquid form, reducing methane release, and can lead to a 60%–90% reduction in greenhouse gas emissions compared to conventional methods^145^. Processes such as pyrolysis and gasification for the extraction of valuable metals such as gold and aluminum (requiring temperatures of around 600 °C), production of zeolite (at 500 °C), or phosphorous recovery (at 600 °C to 850 °C) ensure that even the most resistant contaminants such as PFOS, nickel, chromium, and mercury are fully degraded^146^. These high-temperature novel processes manage sewage sludge safely and recover resources, thereby offering an environmentally friendly solution to waste management^146^.

Additional applications, such as metal recovery at low temperatures, cellulose recovery, or enzyme production, do not involve high-temperature treatments but use chemical processes that can potentially dissolve or deactivate contaminants^147,148^. These applications serve as a valuable preliminary treatment step before integrating the sludge into other applications such as construction materials. Alternatively, these applications can help achieve a higher quality classification (upgrading from grade C to grade A biosolids), yielding sewage sludges qualified for unrestricted use in agriculture^149^. Therefore, these approaches add value by potentially reducing the contaminant load and improving the safety of subsequent applications.

#### Future directions for research on sewage sludges

This perspective aims to promote a new line of inquiry into alternative applications of sewage sludges while describing relevant early-stage developments. Our examination of sewage sludges’ innovative applications, particularly in construction materials, enzyme production, and material recovery, stands as a testament to the value-added applications of this resource. While this review mainly focused on non-energy applications, it should be noted that renewable energy is also produced from sewage sludge. This is mainly done through anaerobic digestion (AD) in which organic matter of sewage sludge is converted into a mixture of methane and carbon dioxide. The methane content in the biogas can be upgraded^150^ and injected into natural gas pipelines, used or converted into electrical power for direct use; however, a significant challenge in this process is managing the high levels of nitrogen and phosphorus in the wastewater byproduct, known as “centrate.” Among various methods to address this issue, acidophilic algal treatment of AD centrate has emerged as an effective option^151^. Acid-loving algae effectively absorb excess ammoniacal nitrogen as NH4+ and phosphorus. Treating centrate this way avoids the parasitic costs of mixing centrate with incoming wastewater as is currently done. Algal biomass can be processed into a variety of products, including animal feed, high-value pigments^152^ bio-oil, biofuels, bio-asphalt, and other bioproducts. The efficacy of acidophilic algal treatment of centrate by the mixotrophic species Galdieria sulphuraria effectively remove bacteria and viruses^153–155^. Cyanidioschyzon merolae is highly compatible with G. sulphuraria and these algae are known to be the dominant microbes present during low pH wastewater treatment^156^. Critically, strains of both organisms can be readily modified via genetic engineering^157,158^. These strains and technologies provide the unique opportunity to combine metabolic with wastewater treatment to create sustainable bio-refineries. The potential of sewage sludges as a pivotal component in sustainable development is largely untapped. Recognizing this potential necessitates a reevaluation of prevailing waste-management paradigms and advocating a transition toward more innovative and sustainable methodologies. Integrating sewage sludges into the circular-economy framework not only addresses the critical issue of waste accumulation but also aids in the conservation of natural resources and the mitigation of environmental impacts. As a step toward this vision, the following research is proposed:

- Advance technological innovation: develop and optimize technologies for converting sewage sludges into valuable products. This includes exploring novel methodologies to extract and refine materials from sewage sludges and to improve the quality and performance of products derived from sewage sludges.
- Ensure safety and environmental integrity: given the complex composition of sewage sludges, ensuring the safety of biosolid applications is paramount. Future studies must rigorously assess the potential health and environmental risks associated with applications of sewage sludges. This entails comprehensive risk assessments, long-term monitoring, and the development of stringent safety standards and guidelines.
- Expand life-cycle assessments (LCAs): conduct comprehensive LCAs to validate the environmental sustainability of biosolid applications. Future LCAs should encompass the entire lifecycle of products derived from sewage sludges, from the generation and processing of sewage sludges to the use and disposal of products.
- Perform research on policy support and regulatory frameworks: to advance the use of sewage sludges, it is essential to have a robust body of research that can help policymakers and regulatory bodies understand and develop policy and regulatory frameworks. Future studies should focus on analyzing how policies and regulations can be improved to promote safe, sustainable, and innovative use of sewage sludges while also protecting public health and the environment.
- Perform research on means of public engagement: the success of biosolid applications heavily relies on public perception and stakeholder engagement. Future research should focus on methods to improve public understanding and acceptance of new applications for sewage sludges, evaluate the impact of educational campaigns on community perceptions, and investigate the role of stakeholder involvement in the development and implementation of innovative biosolid applications.

## Conclusion

This review has articulated the potential of innovative, sustainable methodologies for the application of sewage sludges beyond their conventional uses such as land applications. Through a critical examination of emerging alternatives in materials recovery, construction applications, and enzyme production from sewage sludge, this discourse contributes to the evolving narrative on strategies for sustainable waste management. These proposed applications advocate for a shift toward integrating sewage sludges into models for a circular economy, emphasizing resource efficiency and environmental protection.

Although the possibilities described here are promising, the transition to sustainable applications of sewage sludges requires a commitment to evidence-based research that fully accounts for environmental impacts. Currently, life-cycle assessments (LCAs) for most of these new biosolid applications are still in the early stages and necessitate comprehensive examination. Conducting detailed LCAs is crucial to determining the environmental, societal, and economic consequences of these applications. Integrating these assessments into the research framework provides the necessary insights and decision support systems to promote sustainable practices.

This perspective aims to open a dialogue to encourage a new line of inquiry into alternative applications for sewage sludge, providing examples and highlighting newly developed applications for sewage sludges beyond land applications. The implications of this research extend beyond academic discussions, offering practical ways for policy development, industry practices, and environmental stewardship in the management of sewage sludges.

## Data Availability

All relevant data are available upon request from the corresponding author.
